# Earth’s oldest ‘Bobbit worm’ – gigantism in a Devonian eunicidan polychaete

**DOI:** 10.1038/srep43061

**Published:** 2017-02-21

**Authors:** Mats E. Eriksson, Luke A. Parry, David M. Rudkin

**Affiliations:** 1 Department of Geology, Lund University, Sölvegatan 12, SE-223 62 Lund, Sweden; 2 Life Sciences Building, University of Bristol, 24 Tyndall Avenue, Bristol BS8 1TH, UK; 3 Department of Earth Sciences, Natural History Museum, Cromwell Road, London SW7 5BD, UK; 4 Department of Natural History, Royal Ontario Museum, 100 Queen’s Park, Toronto, Ontario, M5S 2C6, Canada

## Abstract

Whilst the fossil record of polychaete worms extends to the early Cambrian, much data on this group derive from microfossils known as scolecodonts. These are sclerotized jaw elements, which generally range from 0.1–2 mm in size, and which, in contrast to the soft-body anatomy, have good preservation potential and a continuous fossil record. Here we describe a new eunicidan polychaete, *Websteroprion armstrongi* gen. et sp. nov., based primarily on monospecific bedding plane assemblages from the Lower-Middle Devonian Kwataboahegan Formation of Ontario, Canada. The specimens are preserved mainly as three-dimensional moulds in the calcareous host rock, with only parts of the original sclerotized jaw walls occasionally present. This new taxon has a unique morphology and is characterized by an unexpected combination of features seen in several different Palaeozoic polychaete families. *Websteroprion armstrongi* was a raptorial feeder and possessed the largest jaws recorded in polychaetes from the fossil record, with maxillae reaching over one centimetre in length. Total body length of the species is estimated to have reached over one metre, which is comparable to that of extant ‘giant eunicid’ species colloquially referred to as ‘Bobbit worms’. This demonstrates that polychaete gigantism was already a phenomenon in the Palaeozoic, some 400 million years ago.

Polychaete worms comprise one of the most abundant and diverse invertebrate groups in modern oceans, occupying habitats from beach environments to abyssal plains^1^. They range from millimetre-sized parasitic, pelagic and interstitial forms to gigantic benthic representatives measuring some metres in total body length^1^,^2^. They vary considerably in mode of reproduction and feeding habits, encompassing sessile filter feeders through agile hunters^1^,^2^,^3^.

Despite their primarily soft anatomy and resulting low preservation potential^4^, the fossil record of polychaetes extends to the beginning of the Palaeozoic. Recorded occurrences include a wide array of preservational modes, ranging from whole bodies with soft tissues, through abundant calcareous tubes of serpulids from the mid-Triassic or putatively the Permian onwards^5^, to isolated sclerotized jaws (scolecodonts) and jaw apparatuses. The oldest stem group annelids are of early Cambrian age, with full body carbonaceous compression fossils known from the Sirius Passet Lagerstätte of Greenland^6^,^7^,^8^,^9^, and the Guanshan biota in China^10^. The best-known and most abundant Cambrian polychaetes, however, are from the Burgess Shale Lagerstätte of British Columbia^11^,^12^.

Despite their relative rarity compared to many other fossil groups, the fossil record unambiguously shows that polychaete annelids, like today, were an abundant and diverse group of invertebrates in ancient oceans. Crucially, the fossil record reveals long extinct body plans, such as the armoured machaeridians^13^, and shows that some extant groups had a much higher diversity in the past. The latter is the case for polychaetes of the order Eunicida, in which there are 15–20 known fossil families compared with only seven today^1^,^14^. This diversity is demonstrated by scolecodonts, which can be extracted in large numbers from ancient sea floor sediments, particularly by acid maceration of rocks. As well as Eunicida, sclerotized jaws are also produced by the Phyllodocida, and in both groups these are utilized in feeding and in manipulating the environment (e.g., excavating burrows in the substrate). Despite Eunicida and Phyllodocida being closely related (and probably sister taxa), based on analyses using morphological^15^ and molecular^16^ data, the jaws of the two groups function differently; they also differ in composition^1^,^17^,^18^, grow continuously in Phyllodocida and by moulting in Eunicida^19^, and consequently are probably not homologous.

Whereas phyllodocidans first appeared in the Middle Devonian or possibly earlier, and have a rather meagre fossil record, eunicidans are present in the latest Cambrian and are common from the Middle Ordovician onwards^20^. Eunicidan scolecodonts are particularly well-known in marine microfossil assemblages of the Early-middle Palaeozoic^14^,^20^,^21^,^22^,^23^,^24^,^25^,^26^.

Although Ordovician and Silurian jawed polychaete faunas are better known than those of the remainder of the Palaeozoic^24^, the Devonian seems to have been an important time in polychaete evolution. In addition to the putative appearance of phyllodocidans, new characteristic eunicidan forms appeared, such as kielanoprionids and their allies^8^,^22^,^26^,^27^,^28^.

Here we describe and discuss an extraordinary new taxon from the Devonian of Canada. The jaws of this taxon achieve a remarkable size and are the largest known fossil scolecodonts from any time period. Investigation of the relationship between body and jaw size suggests that this animal achieved a body length in excess of a metre and thus is the largest known fossil eunicidan annelid and represents a unique case of ancient polychaete gigantism.

## Geology and Age

The study material is of Late Emsian-Early Eifelian (Early-Middle Devonian) age and derives from the Kwataboahegan Formation^29^,^30^,^31^,^32^ of the Hudson Platform, Hudson Bay Basin (Moose River Satellite Basin) Ontario, Canada. All specimens were collected at Rabbit (Askaskawayau) Ridge, approximately 11 km southeast of Moosonee townsite, centred on N51°11′ W080° 30′ (Canada NTS 1:50 000 42 P/2: Bushy Island, Cochrane District). The area was accessed by helicopter from Moosonee and limited surface sampling was undertaken over several hours on a single day. Less than 0.5 m of section are exposed at this isolated outcrop, which is developed in shallow gravel extraction scrapes exploiting Holocene raised beach ridges. Excavation has revealed glacially scoured bedding planes in limestones comprising thin- to medium-bedded, peloidal bioclastic wackestones to packstones with fossiliferous bioclastic grainstone interbeds. The Rabbit Ridge grainstones yield a diverse, well-preserved assemblage of trilobites representative of the *Paciphacops*-Proetid Association recognized at other Kwataboahegan Formation sections in the Moose River Basin^33^. Overall, the lithologies and fossils here are similar to those in the upper 3 m of a 10 m section in the Labelle Quarry south of the Moosonee townsite, about 15 km to the southwest^34^.

Kwataboahegan Formation is the most conspicuously fossiliferous Devonian unit in the Moose River Basin and contains a macrofauna dominated, in abundance and diversity, by rugosan and tabulate corals, stromatoporoids, brachiopods, and molluscs^35^, with trilobites comprising an important secondary component^33^. The documented microfossil record includes spores^36^, acritarchs^37^, and conodonts^29^, used also for age determinations. Scolecodonts are noted, but not identified, in earlier regional reports^35^ and more recently in studies on thermal maturation^38^.

Deposition of the largely subtidal carbonate succession took place in a shallow shelf and, in the lower part of the unit, coral-stromatoporoid biostromes and bioherms are developed locally over topographic highs that reflect underlying basement relief. Away from these relatively massive skeletal build-ups, the Kwataboahegan is generally thinner bedded and more bituminous.

## Materials and Methods

The scolecodont material is preserved as bedding plane specimens and includes three rock slabs with semi-articulated clusters. The specimens are preserved in a pale beige-greyish mud/wackstone host rock in which cm-sized rugosan corals also occur. Most jaw elements are preserved as (negative) moulds whereas in some specimens parts of the original organic jaw walls are still present. Many specimens show some *post mortem* deformation, and where original jaw walls are preserved those are usually brittle and fragmented. However, the assemblages seem to have undergone relatively little *post mortem* transport; some paired elements are preserved together or in close proximity, which also facilitates assessment of general jaw apparatus architecture.

The material is housed in the Invertebrate Palaeontology Section, Department of Natural History at the Royal Ontario Museum, Toronto, Canada (repository; ROM, followed by digits). This published work and the nomenclatural acts it contains have been registered in ZooBank (http://zoobank.org/urn:lsid:zoobank.org:pub:54C9B58F-1F96-4543-BCC8-D0D7FFEBCBD1).

Specimens were photographed using a Canon EOS 550D digital camera, with a EFS 60 mm f/2.8 Macro USM objective, mounted on a table set-up with four external light sources, and through a Olympus SZX16 microscope equipped with an Olympus SC30 digital camera and operated by cell Sens Standard software.

Micro-CT scanning, using a Nikon HMX ST 225 system, housed at the University of Bristol, UK, was employed and allowed detection of additional scolecodonts concealed in the host rock. 3141 projections were collected for each scan and reconstructed using a modified Feldkamp back projection algorithm^39^ in CT Pro (Nikon Metrology, Tring, UK). The data were then visualised using volume rendering in Drishti^40^.

**Systematic Palaeontology**

Phylum ANNELIDA Lamarck, 1809

Subclass ACICULATA Rouse & Fauchald, 1997

Family Incertae Familiae

*Discussion*. The family affinity of the new taxon described below remains uncertain for the time being (see also species remarks below)

Genus WEBSTEROPRION gen. nov.

*Type and only species. Websteroprion armstrongi* gen. et sp. nov.

*Diagnosis*. Asymmetrical jaw apparatus with maxillae that can grow to >10 mm in size; forceps-like, sub-symmetrical, denticulated MI, with prominent fang and anteriorly spaced, large denticles; MII with shank representing c. 1/2 of jaw length, anteriorly paucidentate dentary and pointed, sub-triangular ramus. Denticulated, short, sub-triangular basal plate.

*Discussion*. In addition to the diagnosed elements, some anterior maxillae and accessory elements were recorded (see description).

*Etymology*. Named after Alex Webster – a ‘giant’ of a bass player – combined with ‘prion’ meaning saw.

**Figure 1:**
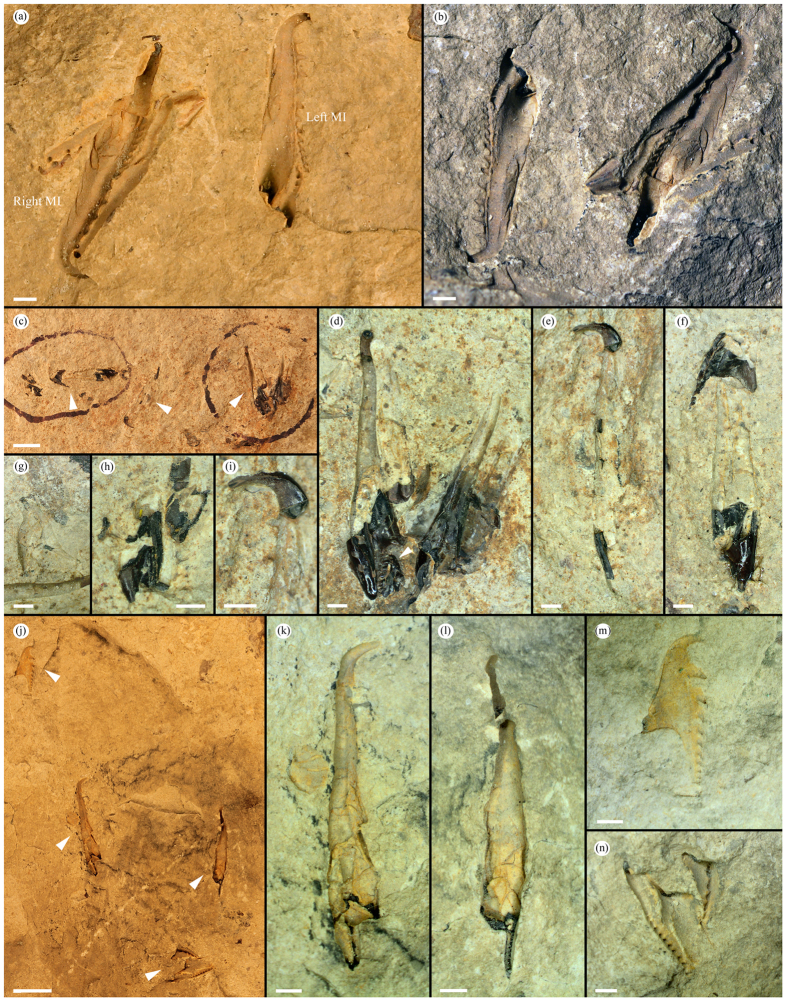
Photographs of *Websteroprion armstrongi* gen. et sp. nov. (**a**) Cluster ROM63122, holotype, bedding plane specimens preserved in dorso-ventral view as negative moulds (except for small black pieces of original jaw wall, see posteriormost tip of right MI); (**b**) Same specimen (ROM63122) flipped 180 degrees and with alternate lighting making the specimens appear positive (as an alternative to making peels with the risk of damaging the fragile specimens); (**c**–**i**) ROM63121; (**c**) Slab overview with white arrows indicating specimens enlarged in (**d**–**i**); (**d**) Part of cluster, note basal plate situated in bight of right MI (indicated by arrow); (**e**) Right MI in lateral view. (**f**) Fragmentary right MII in dorsal view and right(?) MI in ventro-lateral view; (**g**) Imprint of MII (next to MI in (**d**)); (**h**) Crushed maxillae; (**i**) Fang with cutting edge of right MI (same as in (**e**)); (**j**–**n**) ROM63120; (**j**) Slab overview with white arrows indicating specimens enlarged in (**k**–**n**); (**k**) Right MI; (**l**) Left MI; (**m**) Right MII in ventral view; (**n**) Unknown maxillae and to the right an anterior maxilla. Scale bars 1 mm except (**c**,**j**) 5 mm.

**Figure 2:**
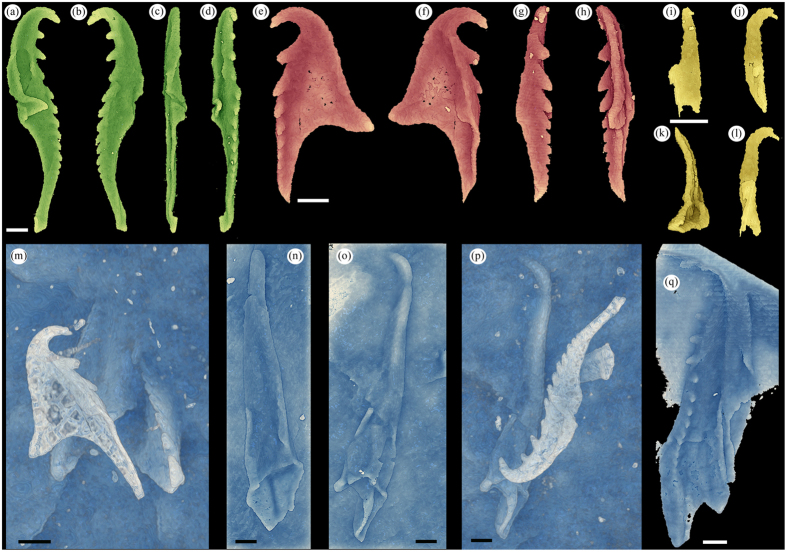
*Websteroprion armstrongi* gen. et sp. nov. CT-scanning reconstructions of specimens detected on (**n,o**) and concealed within (remaining figures) slab ROM 63120; (**a**–**d**) Laterally strongly compressed left MII in (**a**) left lateral, (**b**) right lateral, (**c**) ventral, (**d**) dorsal view; (**e**–**h**) Right MII (posteriormost tip might be missing) in (**e**) dorsal, (**f**) ventral, (**g**) left lateral, (**h**) right lateral view; (**i**–**l**) Lateral or intercalary tooth in different views; (**m**) Right MII (same specimen as in (**e**–**h**)) on top of unknown element and an anterior right maxilla; (**n**) Left MI in ventral view (same specimen as in Fig. 1l); (**o**) Right MI in ventro-lateral view (same specimen as in Fig. 1k); (**p**) Right MI (same specimen as in (**o**)) and left MII (same specimen as in (**a**–**d**)); (**q**) Right MI in dorsal view. Scale bars 1 mm.

**Figure 3:**
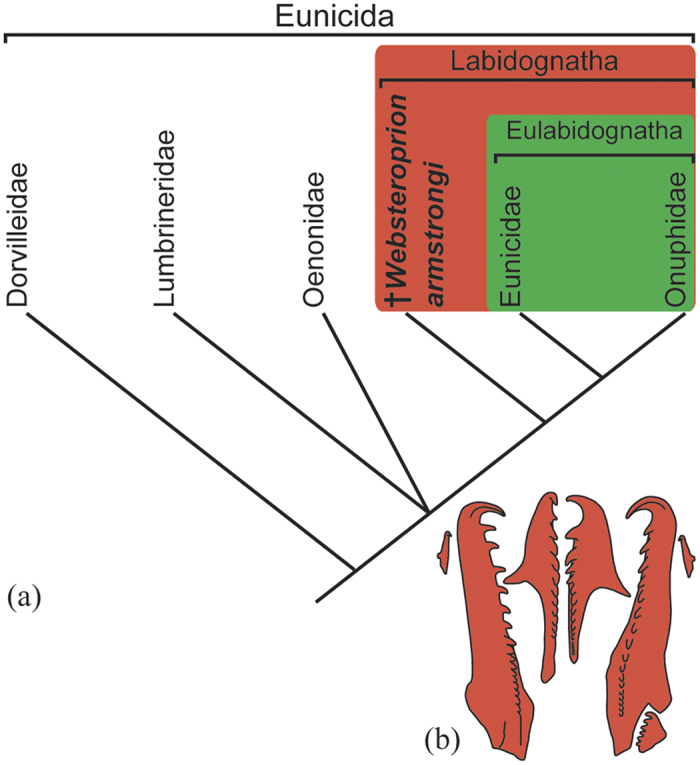
(**a**) Phylogenetic position of *Websteroprion armstrongi* gen. et sp. nov., based on the discussion in Paxton^44^; (**b**) Schematic drawing of the dorsal maxillary apparatus of *W. armstrongi*, showing the main elements.

*Websteroprion armstrongi* sp. nov. (Figs 1,2 and 3)

*Diagnosis*. As for genus.

*Description. Right MI, dorsal view*. Length 11.1–13.5 mm (note that specimen in Fig. 2q is incomplete and might be even larger than this size spectrum), width c. 1/4 to slightly less of jaw length; maximum width in posterior 1/3 of jaw. Strongly elongate jaw tapering anteriorly and terminating in well-developed, gently curved and dorso-laterally directed fang (or hook) that is nearly circular in anterior cross-section. Distinct cutting edge visible on outer face of fang when original jaw wall is preserved (Fig. 1i). Posterior part of jaw sub-rectangular and bulky (Figs 1d and 2q). Inner margin runs nearly straight posteriorly and sub-parallel to outer margin but bends gently inwards at approximately mid-length only to continue posteriorly and terminating in short shank with postero-sinistal directed end. Shank represents 1/5 or less of jaw length. Inner wing thin to insignificant. Posterior jaw termination characterized by sub-triangular bight for fitting of basal plate. Relatively deep basal furrow, widest in posterior and tapering off anteriorly. Dentary, which consists of c. 15–17 sub-conical and posteriorly slanting denticles, occupies anterior c. 0.75 or more of jaw length (fang included). Anteriorly, dentary situated along inner margin, posteriorly from c. 1/4 it curves gently onto slightly elevated ridge on dorsal face and continues sub-parallel to inner margin. Anteriormost 6 denticles relatively large, evenly distributed and paucidentate. Posterior remaining denticles smaller, more tightly packed, and gently and evenly decrease in size posteriorly, ending at undenticulated ridge, transition to which is indistinct in specimens at hand (which hampers precise measurement of extension of dentary). Outer margin runs almost straight posteriorly from fang; at approximately mid-length curves gently outwards, bends and continues postero-dextrally and then antero-laterally into bight; giving rise to characteristic, sub-angular ramus. In ventral view precise morphology of myocoele opening difficult to assess but seems strongly enclosed, representing c. 0.22 of jaw length, based on CT data (Fig. 2o).

*Left MI, dorsal view*. Jaw similar to mirror image of right MI with some features differing, particularly posteriorly. Length 10.0–13.2 mm, width c. 1/4 of jaw length; maximum width in posterior c. 1/4–1/5 portion. Strongly elongate, sub-triangular jaw tapering anteriorly and terminating in well-developed, gently curved and dorso-laterally directed fang (or hook). Inner margin runs nearly straight and sub-parallel to outer margin posteriorly from falcal arch. Posteriormost jaw termination lacks bight and is transversely cut forming a nearly straight to slightly undulating posterior margin with basal angle of c. 35° against length axis. Paucidentate dentary, housing 14–16(?) rather large, gently posteriorly slanting, sub-conical and evenly spaced denticles spread near inner margin in anterior portion of jaw. Posteriorly dentary seems to move inwards onto dorsal face and slightly further away from inner margin. Anteriormost denticle, c. 0.5 of fang length, followed by second, usually largest, denticle. Posteriorly, denticles decrease very gently and evenly in size. Posteriormost denticles tightly packed, elevated on ridge, which anteriorly becomes very discrete. As for right MI transition to undenticulated ridge difficult to discern. Based on CT data (Fig. 2n) posterior c. 1/6 part of jaw characterized by short, anteriorly rounded sub-triangular inner wing, tapering posteriorly. Similar ‘outer wing’ present on opposite side. Distinct but short basal furrow left of posterior denticulated ridge. Outer margin subparallel to inner margin anterior of outer wing and continues to form part of fang. In ventral view, based on CT-data, myocoele opening is strongly enclosed (Fig. 2n), representing c. 0.2 of jaw length.

*Right MII, dorsal view*. Length 6.6–6.8 mm, width c. 0.45 of jaw length. Dentary arranged along inner margin in straight to vaguely and convexly curved fashion, holding 19 sub-triangular, posteriorly slanted denticles (including cusp). Relatively large sickle-shaped, single cusp pointing laterally and slightly dorsally, followed by 2–3 widely spaced, sub-triangular denticles. Following these are normal-sized denticles that rapidly decrease in size and become more tightly packed posteriorly. Posteriormost 7–8 denticles extremely small and tightly packed (see broken off posteriormost extremity; Fig. 1m). Shank occupying c. 1/2 of jaw length with sub-parallel sides and tapering slightly posteriorly. Bight shallow, anteriorly straight to slightly concave; bight angle near 90°. Ramus moderately long, terminating laterally in sub-triangular extremity (partly concealed in Fig. 1m). Undenticulated ridge insignificant. Anterior outer margin straight to slightly sigmoidal, curves and continues into cusp. Inner wing insignificant. In lateral view dentary straight and maximum jaw width at approximately mid-length. In ventral view extension of myocoele opening difficult to discern but seemingly follows anterior part of ramus across jaw, thus representing slightly more than 1/2 of jaw length (Fig. 2f).

*Left MII, dorsal view.* Only one, laterally strongly compressed, left MII was identified using CT-scanning, i.e., element fully concealed in host rock (Fig. 2a–d). Length c. 10.8 mm, measured width 0.12 of jaw length (but estimated as >0.25 of length in laterally uncompressed elements); maximum width at tip of ramus. Elongate element with dentary arranged on elevated ridge, particularly pronounced in middle of jaw, close to outer margin. Moderately large, postero-dorsally curved single cusp followed by 11(?) denticles that decrease evenly and gently in size posteriorly. First 3 post-cuspidal denticles rather widely spaced, posteriorly denticles become more tightly packed. Transition to undenticulated ridge indistinct and minute denticles might be present in posteriormost c. 1/4 of jaw, otherwise this part is undenticulated. Triangular and pointed ramus with straight posterior margin; bight represents c. 0.47 of jaw length; shank long, slender tapering posteriorly. In lateral view jaw gently sigmoidally curved. In ventral view extension of myocoele opening difficult to discern but seemingly follows anterior part of ramus across jaw, thus representing c. 0.49 of jaw length.

*Anterior maxillae*. Cluster 63120 (Fig. 1n) hosts partial anterior element, possibly right MIV. Sickle-shaped as preserved with c.7 denticles decreasing in size posteriorly. CT-scanning revealed another anterior element (Fig. 2m), possibly right MIV; Posteriorly bent cusp followed by diastema and 6 (visible) rather tightly packed denticles, seemingly 1/2 cusp length. Ramus seems short and anteriorly situated.

*Basal plate*. Small, sub-triangular (partly crushed) element situated in bight of right MI in ROM 63121 (Fig. 1d). Representing c. 0.17 of jaw length of right MI this element holds 6 relatively large, conical denticles of near equal size, distributed along straight inner margin. Outer margin runs straight postero-dextrally from cusp, bends sharply at approximately mid-length and continues towards, and finally merges with, posteriormost part of dentary, giving rise to overall sub-triangular element outline.

*Lateral/intercalary tooth.* CT-scanning revealed one small element probably representing lateral or intercalary tooth (Fig. 2i–l); its relative size and morphology correspond well to remaining maxillae. Length c. 2.6 mm, width c. 0.3 of jaw length; maximum width in posteriormost 1/3 of jaw. Sub-conical, slender and vaguely curved element tapering towards and terminating with pointed, dorsally bent apex.

*Etymology*. Named after Derek K. Armstrong, who found and collected the first specimens in the field.

*Holotype.* ROM63122 (Fig. 1a,b); incomplete jaw apparatus, collected July 7, 1994. Remaining illustrated specimens (ROM63120 and ROM63121; Figs 1 and 2) designated paratypes.

*Occurrence*. Upper Emsian-Lower Eifelian Kwataboahegan Formation at Rabbit (Askaskawayau) Ridge, Ontario, Canada.

*Remarks*. Despite difficulties in determining the full morphology of the jaw apparatus and individual jaws because of the state of preservation, this unambiguously represents a new taxon based on the unique combination of characters. It cannot be excluded that the element designated as the left MII might constitute a single left MIII. Most likely *W. armstrongi* deserves the establishment also of a unique family; however, pending the discovery of additional material, it is left in open nomenclature at the family level.

Whereas the combination of element morphologies is unique for *Websteroprion*, the MI and MII in particular share some characters with those of hadoprionids, paulinitids, ramphoprionids, and kielanoprionids^21^,^41^,^42^,^43^. For example, whilst the MI of hadoprionids are reminiscent of those of *Websteroprion* in being elongate and sturdy with anterior hooks and paucidentate dentary, the remaining anterior maxillae are very different with their long, straddling denticles. The MI of paulinitids are more forceps-like and dorso-ventrally flattened and the right MI has a shallower bight with a fused, small basal plate compared to that of *Websteroprion*. The paulinitid MII are, however, similar in general appearance to those of *Websteroprion*. Among ramphoprionids the similarities pertain to the larger-sized genera *Ramphoprion* and *Megaramphoprion*, with which *Websteroprion* shares the prominent, elongate MI with a posteriorly truncated termination of the left MI and also, to some extent, the dentary. The anterior dentary of the MI of kielanoprionids can be paucidentate, resembling that of *Websteroprion*. However, the elements are overall bulkier and, similar to those of paulinitids, have a dentary that is more inwards projected, or facing the opposing element. Moreover, the right MI of kielanoprionids lacks a distinct bight and basal plate.

Thus *W. armstrongi* is intermediate also between the eulabidognath and labidognath type of jaw apparatus architecture *sensu* Paxton^44^. Additional jaw elements (particularly the carriers), and in better state of preservation, are needed in order to unambiguously designate the apparatus type. However, the denticulated and unfused basal plate and accessory element (putative intercalary/lateral tooth) suggest that *W. armstrongi* belongs to the labidognath type (Fig. 3a,b). Extant eulabidogaths, Onuphidae and Eunicidae, are generally accepted as descending from the extinct family Paulinitidae^21^,^45^, an ancestral eulabidognath *sensu* Paxton^44^.

It is noteworthy also that the MI of *W. armstrongi* is similar to the larval MI of extant onuphids and eunicids (see Paxton & Safarik^46^, Fig. 1F; Paxton & Eriksson^47^, Figs 1b and 3). Whilst adult onuphids and eunicids possess an undeticulated MI^46^, the larval left MI possesses prominent denticles and a similar gross morphology to the MI of *W. armstrongi.* Thus the appearance of the larval MI of these two modern eunicidan families appears to recapitulate the ancestral adult morphology^47^, which also strengthens the case of *Websteroprion* being related to the extant Eunicidae and Onuphidae (Fig. 3).

The serendipitous discovery of multiple specimens from a monospecific assemblage in limited sample volume may suggest that *W. armstrongi* was a common species at this particular locality of the Kwataboahegan Formation. The specimens likely represent ‘snap-shot assemblages’ of deceased individuals that were rapidly entombed by sediment and subjected to limited transport. It is, however, puzzling that no mandibles (which also are expected to have been huge) were found associated with the maxillary clusters. This might be related to structural and/or slight biochemical differences that were less suitable for preservation in this particular environment. Alternatively, this indicates that the maxillae represent shed specimens and the animal (with the continuously growing mandibles) died elsewhere.

*W. armstrongi* adds to the list known Devonian polychaete taxa and also suggests that eunicidan diversity and disparity were high during this time.

### Feeding habits

*Websteroprion armstrongi* was a raptorial feeder *sensu* Fauchald & Jumars^3^. Such polychaetes use their buccal apparatus, consisting of a muscular ventral or axial pharynx, to snatch food items^1^,^3^. Eunicidans and phyllodocidans use their jaws to capture live animals as carnivores, to rip off pieces of algae, as herbivores, or to grasp dead and decaying organic matter, as scavengers. Although it would perhaps be easy to assume that *W. armstrongi* had a predatory, carnivorous mode of feeding based on the ‘fierce’ appearance of the jaws, it has been shown that extant jaw-bearing polychaetes exhibit a wide range of feeding habits^1^,^3^ and, thus, that jaw morphology does not necessarily reflect specific modes of feeding. Moreover, in many active predators antennae and palps are present on the prostomium^1^ and such soft-body features are obviously not known for *W. armstrongi*, although they are assumed to have been present based on their presence in the extant eulabidognaths Eunicidae and Onuphidae^2^. Therefore, without evidence of preserved gut content and/or soft body structures, more precise knowledge of the feeding habits of *W. armstrongi* remains elusive for the time being.

### Size estimates of *Websteroprion* and polychaete gigantism

The jaws of *Websteroprion* are colossal in size compared to most scolecodonts known from the fossil record and also compared to the jaws of most extant aciculates. Scolecodonts are typically found in the size range of 0.1–2 mm, although exceptions beyond both ends of this spectrum exist. Only some 30 specimens are known from the fossil record – distributed primarily among ramphoprionids, paulinitids, polychaetaspids, hadoprionids, and atraktoprionids – with a size of 3.5 mm (arbitrarily chosen upper limit) or more (electronic Supplementary Material, Table S1). By contrast, the MI of *W. armstrongi* reach >13 mm in length. In the published literature (Table S1) there is an interesting record of one partial specimen, described by Eller^48^ as *Arabellites longiformis* from the Devonian of Ontario Co., New York. Eller measured the specimen to 8 mm and estimated that if intact it could have reached 14 mm in length. Based on the rudimentary drawing it shows similarities to the (anterior part of) the MI of *W. armstrongi*. However, Eller’s specimen only comprises the anterior portion of an MI which are undiagnostic and usually homeomorphic between many taxa, even at genus and family level. Therefore, as the specimen could belong to any number of taxa, *A. longiformis* should be regarded as a *nomen dubium*.

Based on the fossil record data (Table S1) there seems to be no general trend towards a maximum size increase with time towards the Devonian, suggesting that *W. armstrongi* is a genuine aberration. Whilst it is uncertain if the exceptional size of the jaws of *W. armstrongi* represents pathological or gerontic stages, the presence of multiple individuals of similar size is congruent with the interpretation that attaining large size was characteristic of the species. Size and morphology of the maxillae indicate that the individuals are adults^44^,^47^ and given that that some polychaetes grow continuously throughout life it is possible that adults could have grown bigger still. The absence of smaller individuals may indicate that adults and juveniles had different environmental preferences, as is the case in some extant eunicidans. Nevertheless, it is clear that the maxillae of *W. armstrongi* are the largest known from the fossil record.

In order to assess the full body size for *W. armstrongi* based on the size of its jaws, comparisons with now-living, jaw-bearing polychaetes are needed. This is, however, not an entirely straight-forward task. Firstly, *W. armstrongi* is extinct and the precise phylogenetic relationships of Palaeozoic scolecodont taxa to the modern eunicidan diversity are currently poorly constrained. Secondly, the relationship between the size of the jaws (maxillae and/or mandibles) and the full worm body size (length and/or width) is unclear or ambiguous even from the neontological literature. When the jaw apparatuses in extant worms are at all measured and described in conjunction with the systematic descriptions of soft tissue characters, they are usually related to the number of chaetigers (or setigers) and not necessarily to body length. In many eunicidan polychaetes replacement of the maxillary jaws have been suggested and/or recorded from more or less frequent moulting during ontogeny^19^,^44^. Moreover, the moult increments during continuous shedding are not necessarily consistent within a single taxon, and it may vary between species. For example, Paxton & Safarik^46^ noted that the jaw apparatus growth rate and moult frequency in the onuphid *Diopatra aciculata* slow down with increasing age.

Despite these problems there are some published data that can be used for comparison, in order to acquire an estimate of the body size of *W. armstrongi*. Ieno *et al*.^49^ found a positive correlation between jaw size and body length for the ragworm *Laeonereis acuta*. Extrapolating their results would result in a total body length of *W. armstrongi* in the excess of 4.1 m. By sharp contrast, however, using the data of Brenchley^50^ (p. 308, Table 6) for *Platynereis bicanaliculata* and *Nereis brandti*, results in an extrapolated body length of *W. armstrongi* of 0.42 m and 0.73 m, respectively. This highlights the difficulties in assessing the body length based on jaws using modern examples. Note, moreover, that these examples are phyllodocidans, whose continuously growing jaws are of a different composition than those of the eunicidan *W. armstrongi*.

Among extant eunicidans we find some of the largest and smallest polychaete species known^51^. Particularly within the family Eunicidae and the genus *Eunice* there are taxa commonly referred to as ‘giant eunicids’^52^,^53^,^54^,^55^, but gigantic worms also are found in Onuphidae, the sister taxon of Eunicidae^56^,^57^. Members of the Eunicidae and Onuphidae have jaw apparatuses of eulabidognath type *sensu* Paxton^44^, similar in architecture to that of *W. armstrongi* (Fig. 3). Leland^57^ studied the relationship between the size of the mandibles (which by contrast to the maxillae grow continuously throughout life and thereby form a more reliable aging structure) and body parameters of giant Australian beach worms (species of the onuphid *Australonuphis*) and found a positive correlation. *Australonuphis* mandibles correspond in length approximately to that of the carriers and first maxilla combined (H. Paxton, Sydney, pers. commun., 2016). Thus a conservative conversion for the length of our MI (c. 2/3 of putative mandible size) and using the calculations of Leland^57^ (Figs. 15, 17, Table 3) for *A. teres* and *A. parateres* would result in a massive body length of *W. armstrongi* in excess of 4.8 and 8.3 m, respectively. However, as for the phyllodocidan examples above, these are (perhaps unlikely) extrapolated values and neglect the significant possibility of changes in trajectory in the relationship between mandible length and body length in considerably larger/older specimens.

For the species-rich genus *Eunice*, where the largest forms are found today, there are published claims of individuals reaching 6 m in length^53^,^58^; however, some authors have argued that they are rather in the order of 3 m^54^. These large eunicids have a wide distribution in modern tropical and temperate seas^55^. The largest species known is the famous ambush predator *Eunice aphroditois* (colloquially and rather infamously referred to as the ‘Bobbit worm’), but it must be emphasized that the taxonomy of this species (or species complex) is controversial and in need of revision^55^,^58^,^59^. For comparison, we received some size data measured (by Luis F. Carrera-Parra, Mexico – pers. commun., 2016) on two giant eunicid species, *E.* ‘*aphroditois*’ and *E. roussaei*, stored at the National Museum of Natural History (Washington DC, USA), in which the jaws are directly related to full body length. In these specimens, an MI length of 0.92–1.2 cm corresponds to body a length of 0.79 to >1.2 m (Table S2). Using these as modern analogues suggests again that *W. armstrongi* could have attained a full body length in excess of one metre.

Although the available data allow for a broad range of inferred sizes, comparison with closely related extant taxa indicates a body length in the region of 1–2 metres. Body sizes much smaller or much in excess of this range are derived either from outgroup taxa with jaws that are not homologous (e.g. Nereididae) or extrapolation from close relatives whose maxillae do not attain such a large size (e.g. *Australonuphis*).

The extraordinarily large jaws and inferred body length of *W. armstrongi* demonstrate that eunicidan worm gigantism had appeared already in the Devonian, some 400 million years ago. Very large body size, or gigantism, in animals is an alluring and ecologically important trait, usually associated with advantages and competitive dominance^60^. It is known among several living clades as well as throughout the fossil record, with an increase in body size trajectory towards the present^61^, but driving mechanism/s are difficult to discern and vary^60^,^61^,^62^. For *W. armstrongi*, it could be related to intrinsic factors (e.g., unique physiology, predation pressure and/or interspecific competition) and/or extrinsic (physical/chemical environmental) factors (e.g., the availably of oxygen, nutrients, food resources) during the time of deposition of the Kwataboahegan Formation in the Rabbit Ridge area. Precise knowledge of the palaeoenvironmental and palaeoecological scenario at Rabbit Ridge is unfortunately limited and the remote locality is difficult to access. By comparison, it is noteworthy that examples of giant conodonts (with elements reaching centimetre-size rather than sub-millimetre-size) are known from unusual environmental (and ecological) settings, now preserved as Lagerstätten; e.g., the Upper Ordovician Soom Shale Lagerstätte of South Africa^63^ and more recently the Middle Ordovician Winneshiek Lagerstätte of Iowa, USA, the strata of which were deposited in a meteorite impact crater^64^. However, while representing an ancient ‘Bobbit worm’ and a case of primordial eunicidan worm gigantism, the specific driving mechanism/s for *W. armstrongi* to reach such a size remains ambiguous.

## Conclusion

Large body size is present in many living soft bodied invertebrate taxa, but establishing if similar sizes were attained in their extinct relatives is often difficult, if not impossible. The specimens of *W. armstrongi* described herein demonstrate gigantism in a group of primarily soft bodied polychaetes, a phenomenon which is also known in its extant closest relatives, but hitherto only among the eulabidognathans Onuphidae and Eunicidae. Thus, this new finding reveals gigantism also in the stem group (the Labidognatha) and that this feature occurs only in one particular clade within the Eunicida (Fig. 3a). This discovery highlights that although comparatively rare, fossils of annelid worms provide important insights into the past diversity, disparity and evolutionary history of the group.

## Additional Information

**How to cite this article:** Eriksson, M. E. *et al*. Earth’s oldest ‘Bobbit worm’ – gigantism in a Devonian eunicidan polychaete. *Sci. Rep.* **7**, 43061; doi: 10.1038/srep43061 (2017).

**Publisher's note:** Springer Nature remains neutral with regard to jurisdictional claims in published maps and institutional affiliations.

## Supplementary Material

Supplementary Material
