# Supplementary material for: Earth’s oldest ‘Bobbit worm’ – gigantism in a Devonian eunicidan polychaete
Source: Sci Rep. 2017 Feb 21;7:43061. doi: 10.1038/srep43061 (PMC5318920; doi:10.1038/srep43061)
Supplement: Supplementary Material [file srep43061-s1.doc]

**Earth’s oldest ‘Bobbit worm’ – gigantism in a Devonian eunicidan polychaete**

**ELECTRONIC SUPPLEMENTRAY MATERIAL**

*Mats E. Eriksson, Luke Parry and David M. Rudkin*

**Supplementary Table S1.**


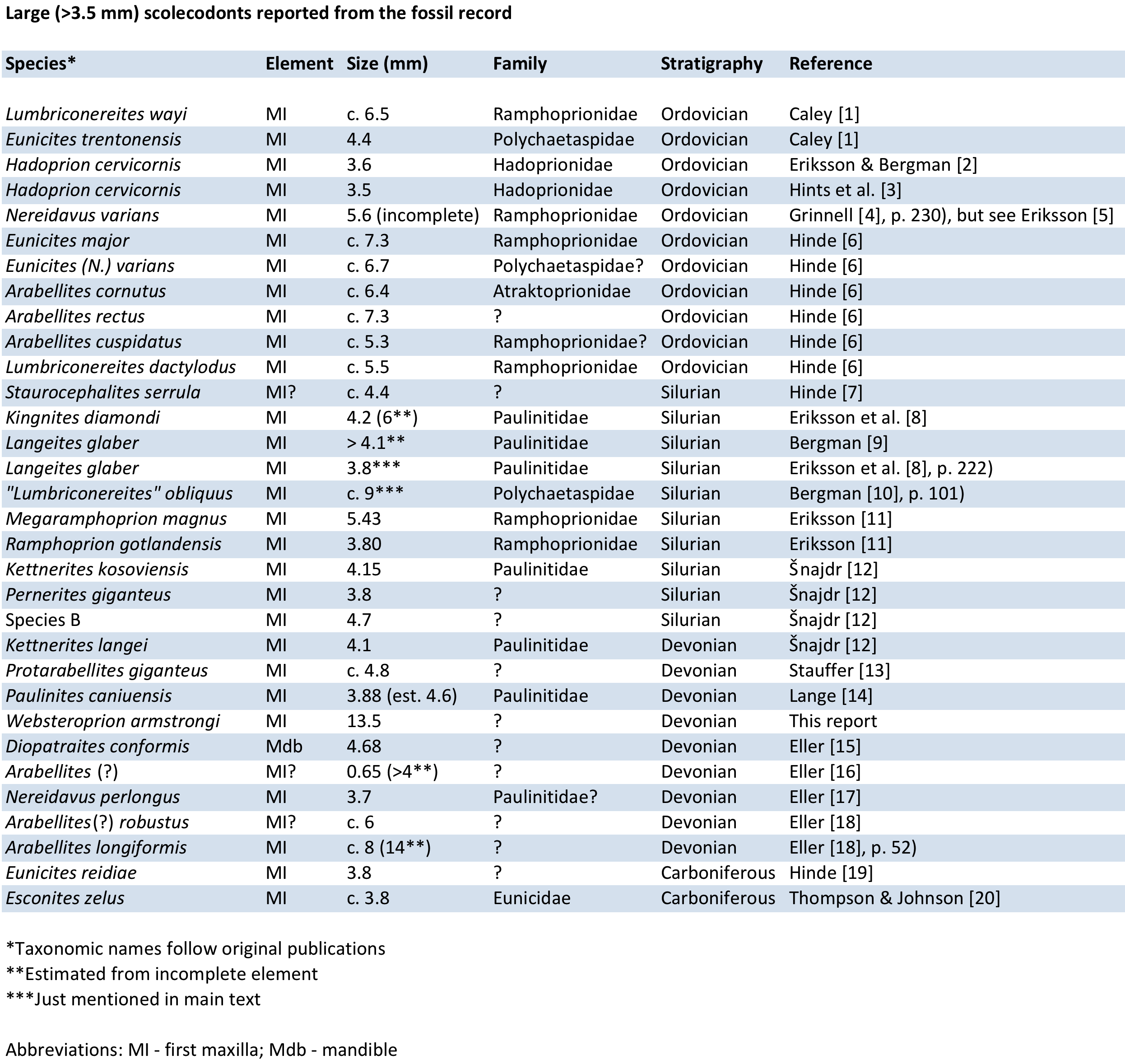


**Supplementary Table S2.**


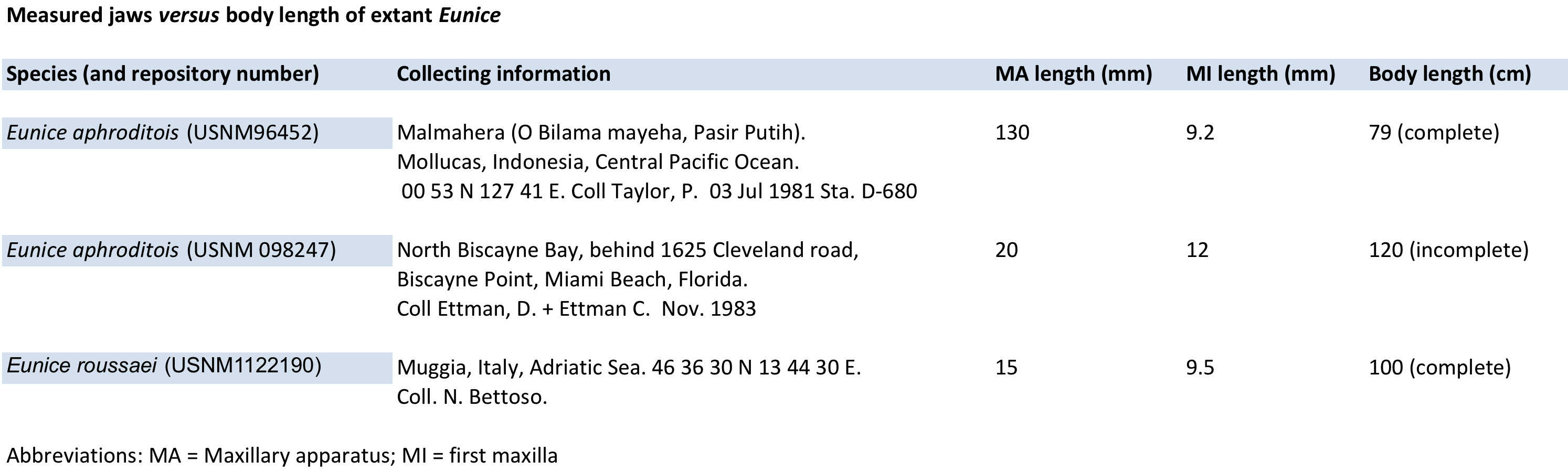


**References in the electronic supplementary material (Table S1)**

1. Caley, J. F. The Ordovician of Manitoulin Island, Ontario. In *Contributions to the Study of the Ordovician of Ontario and Quebec* (eds Wilson, A. E., Caley, J. F., JC Sproule, J. C. & Okulitch, V. J.), 21–95. Geological Survey of Canada Memoir **202**, 1–133. (1936)
2. Eriksson, M. & Bergman, C. F. Scolecodont systematics exemplified by the polychaete *Hadoprion cervicornis* (Hinde, 1879). *J. Paleontol.* **72**, 477–485 (1998).
3. Hints, O., Tonarová, P. & Desrochers, A. Late Ordovician jaw-bearing polychaetes from Anticosti Island, eastern Canada, and their biogeographic significance. *Can. J. Earth Sci.* **53**, 1–8 (2016) (dx.doi.org/10.1139/cjes-2015-0222)
4. Grinnell, G. B. Notice of a new genus of annelids from the Lower Silurian. *Am. J. Sci. and Arts* **14**, 229–230 (1877).
5. Eriksson, M. Taxonomic discussion of the scolecodont genera *Nereidavus* Grinnell, 1877, and *Protarabellites* Stauffer, 1933 (Annelida: Polychaeta). *J. Paleontol.* **73**, 403–406 (1999).
6. Hinde, G. J. On annelid jaws from the Cambro-Silurian, Silurian, and Devonian Formations in Canada and from the Lower Carboniferous in Scotland. *Q. J. Geol. Soc. London* **35**, 370–389 (1879).
7. Hinde, G. J. On annelid jaws from the Wenlock and Ludlow formations of the west of England. *Q. J. Geol. Soc. London* **36**, 368–378 (1880).
8. Eriksson, M. E., Hints, O. & Bergman, C. F. *Kingnites diamondi* gen. et sp. nov., an exceptionally large Silurian paulinitid (Annelida; Polychaeta) from shallow marine settings of Baltoscandia. *GFF* **134***,* 217–224 (2012).
9. Bergman, C. F. Silurian paulinitid polychaetes from Gotland. *Fossils Strata* **25**, 1–128 (1989).
10. Bergman, C. Polychaete jaws. In *Lower Wenlock faunal and floral dynamics – Vattenfallet section, Gotland* (eds Jaanusson, V., Laufeld, S. & Skoglund, R.), 92–102. *Sveriges Geologiska Undersökning* **C762** (1979).
11. Eriksson, M. Silurian ramphoprionid polychaetes from Gotland, Sweden. *J. Paleontol.* **75***,* 993–1015 (2001).
12. Šnajdr, M. O errantnich Polychaetech z českeho spodnihoPaleozoika (= On Errant Polychaeta from the Lower Paleozoic of Bohemia). *Sborník Ústředního ústavu geologického***18**, 241–292 (1951). [in Czech]
13. Stauffer, C. R. Middle Devonian Polychaeta from the Lake Erie District. *J. Paleontol.* **13**, 500–511 (1939).
14. Lange, F. W. Um novo escolecodonte dos Folhelhos Ponta Grossa. *Arquivos do Museu Paranaense Curitiba* **8**, 189–214 (1950). [in Portuguese]
15. Eller, E. R. Scolecodonts from the Potter Farm Formation of the Devonian of Michigan. *Ann. Carnegie Mus.* **27**, 275–287 (1938).
16. Eller, E. R. Scolecodonts from the Windom, Middle Devonian, of western New York. *Ann. Carnegie Mus.* **28**, 323–341 (1941).
17. Eller, E. R. Annelid jaws from the Upper Devonian of New York. *Ann. Carnegie Mus.* **22**, 303–317 (1934a).
18. Eller, E. R. Annelid jaws from the Hamilton group of Ontario County, New York. *Ann. Carnegie Mus.* **24**, 51–56 (1934b).
19. Hinde, G. J. On the jaw apparatus of an annelid (*Eunicites Reidiæ* sp. nov.) from the Lower Carboniferous of Halkin Mountain, Flintshire. *Q. J. Geol. Soc. London* **52**, 448–450 (1896).
20. Thompson, I. & Johnson, R. G. New fossil polychaete from Essex, Illinois. *Fieldiana, Geology* **33**, 471–487 (1977).
